# Contrasting haemosporidian infections in two ecologically distinct wading birds from breeding colonies in the southeastern United States

**DOI:** 10.1016/j.ijppaw.2026.101200

**Published:** 2026-01-27

**Authors:** Ke Zhang, Samantha M. Wisely, Chris K. Gulick, Abby N. Powell

**Affiliations:** a Department of Wildlife Ecology and Conservation, University of Florida, Gainesville, FL, USA; b U.S. Geological Survey, Florida Fish and Wildlife Cooperative Research Unit, Gainesville, FL, USA

**Keywords:** Haemoproteus, Movement ecology, Host–parasite interactions, Threskiornithidae, Ardeidae

## Abstract

Wading birds may serve as ideal hosts for avian hemoparasites, as they are long-lived, undertake extensive movements, form dense breeding colonies, and inhabit water-associated environments that support vectors. Although previous studies have reported parasite species and prevalence in various wading bird species, little is known about their associations with bird behavior and life stage. To address this gap, we examined haemosporidian infections in two ecologically distinct species, white ibis (*Eudocimus albus*) and tricolored heron (*Egretta tricolor*), to explore differences in life stage and movement that may explain prevalence differences. We combined blood screening for hemoparasites with satellite tracking data describing birds’ movement patterns. We screened 95 white ibis (67 juveniles and 28 adults or subadults) and 69 tricolored herons (45 juveniles and 24 adults). We detected a single *Haemoproteus plataleae* lineage in both species, with higher infection prevalence in white ibis (42.1 %) than in tricolored herons (14.5 %). Among white ibis, adults showed a higher prevalence (67.9 %) than juveniles (31.3 %), whereas in tricolored herons, adults had a prevalence of 8.3 % and juveniles 17.8 %. Non-breeding season movement data showed that white ibis used both freshwater and saline habitats across the southeastern United States, which may also serve as habitats for vectors. In contrast, tricolored herons remained mainly along coastal areas, using saline habitats in the southeastern United States and wintering sites in Central America, which may be less favorable for vectors. Overall, white ibis may serve as major reservoirs and sources of reinfection for *H. plataleae*, whereas tricolored herons may facilitate parasite dispersal between breeding colonies along the coasts of the southeastern United States and wintering areas in Central America. This study presents the first direct comparison of hemoparasite infections in two ecologically distinct wading birds and highlights movement data as key to explaining infection differences, providing a baseline for future studies.

## Highlights

• Haemoparasite prevalence was higher in white ibis than tricolored herons at breeding colonies in Alabama, USA.
• White ibis used higher proportions of freshwater habitats than tricolored herons outside of the breeding season.
• Infections occurred across age classes and possibly at both breeding colonies and wintering grounds.
• White ibis may act as parasite reservoirs and support reproduction, whereas herons may transmit parasites across regions.

## Graphical abstract

**Figure:**
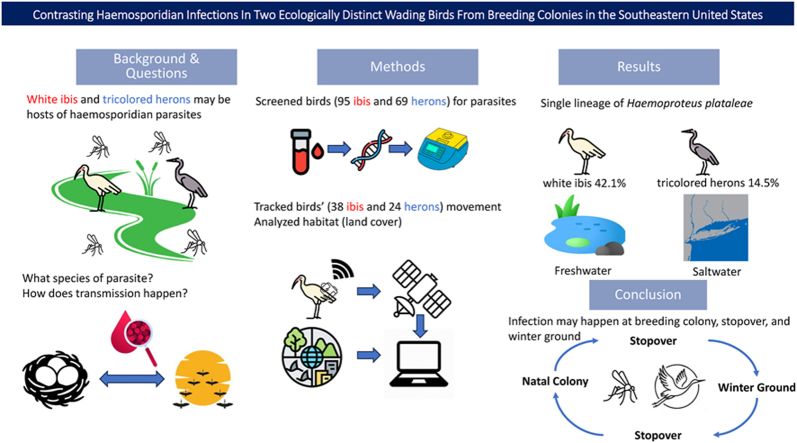

## 1 Introduction

Long-legged wading birds (hereafter referred to as wading birds) include herons and egrets (Ardeidae) and ibises and spoonbills (Threskiornithidae), both within the order Pelecaniformes, as well as storks (Ciconiidae) in the order Ciconiiformes. These species typically form dense breeding colonies and travel long distances across diverse landscapes throughout their lives (Kushlan, 1981; Hafner, 1997). These life-history traits make them potentially ideal hosts for pathogens that cause infectious diseases, as dense breeding colonies facilitate transmission between individuals (Brown and Brown, 2004; Khan et al., 2019), while their wide-ranging movements increase exposure to pathogens across diverse habitats and contribute to their spread across regions (de Angeli Dutra et al., 2021; Yang et al., 2024; Perrin et al., 2025). In particular, wading birds may be important hosts for vector-borne haemosporidian parasites (hereafter “hemoparasites”), which cause avian malaria and related diseases, as they spend much of their time in wetlands and other water-associated habitats where the vectors of these parasites commonly occur (LaPointe et al., 2012).

The most common hemoparasites of birds include *Plasmodium*, *Haemoproteus*, and *Leucocytozoon*, which are distinguished primarily by their morphology and transmission vectors (Valkiunas, 2004). In birds, Hemoparasites typically cause anemia and lethargy, leading to long-term fitness consequences that can persist beyond the initial infection (LaPointe et al., 2012). Infected birds may also experience altered behaviors, such as delayed migration timing (Ágh et al., 2019). Because the effects of hemoparasites on birds are typically chronic and non-lethal, in contrast to acute infections that cause rapid mortality, hemoparasite–host systems provide a valuable model for linking infection patterns with bird behavior. Species from the haemosporidian genera *Plasmodium*, *Haemoproteus*, and *Leucocytozoon* have been detected in wading bird species (Valkiunas, 2004). However, most studies have focused on identifying parasite lineages and reporting infection prevalence rather than examining ecological or behavioral factors potentially influencing these infections (Telford et al., 1992; Coker et al., 2017; Yabsley et al., 2023).

Hemoparasite infections can occur at both breeding and wintering sites, provided that both the host and the vector are available to complete the transmission cycle (Beaudoin et al., 1971). Theoretically, infected birds can serve as hosts, facilitating parasite transmission to vectors and other birds. Long-distance movements, including migration, dispersal, and nomadism, may allow birds to transport parasites between geographically separated populations, potentially introducing infections to naive hosts where competent vectors occur. Under this framework, recent studies have reported mixed results regarding haemosporidian infections in migratory and resident birds. In some studies, migratory populations have shown higher infection prevalence than resident populations. This pattern may reflect the migratory exposure hypothesis, which suggests that long-distance movements increase opportunities for encountering parasites (Altizer et al., 2011), or the migratory susceptibility hypothesis, which suggests that the physiological stress and energetic demands of migration may weaken immune defenses, making long-distance migratory birds more vulnerable to infection (Figuerola and Green, 2000). Conversely, other studies have found lower prevalence among migrants compared to residents, consistent with the migratory culling hypothesis, where heavily infected individuals are less likely to survive migration (Slowinski et al., 2018), or the migratory escape hypothesis, which proposes that migration enables individuals to avoid regions with high parasite transmission (Clark et al., 2016). When applying these hypotheses to wading birds, the situation may become complex. Many wading birds exhibit diverse annual movement strategies, meaning that both resident and non-resident individuals can occur within the same breeding population (Melvin et al., 1999). This makes testing the above hypotheses dependent on a sufficiently large sample size that includes representative individuals of both resident and non-resident individuals, as well as on long-term tracking and continuous sampling. Consequently, hypotheses developed around strictly migratory systems must be reframed to also consider inclusion of non-resident movements. Another challenge is the limited understanding of wading birds’ long-distance movements, such as the connections between breeding and wintering sites. Although an increasing number of studies have used tracking devices to address these knowledge gaps (Picardi et al., 2020; Lim et al., 2021; Huang et al., 2022), such knowledge is still lacking for many wading bird species, and attempts to link these movements with hemoparasites remain limited. As a result, confirming post-breeding movement behavior is an important step toward better understanding haemosporidian transmission in wading birds.

Regardless of whether birds remain as residents or move to distant wintering areas, the habitats they occupy during the non-breeding season also play a critical role in haemosporidian transmission, as these habitats determine their level of exposure to vectors (Isaksson et al., 2013). For waterbirds that rely on aquatic environments, two major habitat types, fresh- and saltwater wetlands, likely differ in vector abundance (Resh, 2009; Ramasamy and Surendran, 2012). In general, freshwater habitats provide more favorable conditions for Dipteran vectors (Keiper et al., 2002), whereas saline environments tend to support fewer suitable vectors (Adler and Courtney, 2019). In some avian species that utilize both freshwater and saltwater habitats, such as shorebirds, it has been hypothesized that birds escape exposure to vector-borne parasites by occupying saline habitats only during the non-breeding seasons (Piersma, 1997). Accordingly, lower levels of haemosporidian infections have been observed in shorebirds occupying saltwater habitats (Figuerola, 1999; Murata, 2002), possibly due to the reduced abundance of vectors in such environments caused by wind, saline, and low vegetation cover (Mendes et al., 2005). For wading birds, the situation may closely approximate that of shorebirds, as some species primarily use freshwater habitats, others use saline environments like estuaries, and some occupy a combination of both habitats primarily during their non-breeding seasons. However, no studies have directly compared haemosporidian infection rates among wading bird species across different habitat types. Furthermore, the situation is even less understood when considering their habitat use during the non-breeding season, as such information is often limited or unavailable for many species (Kushlan, 1981). A clearer understanding of wading bird habitat use, in conjunction with knowledge of vector habitat distribution during the non-breeding period, is therefore beneficial for explaining patterns of haemosporidian transmission and identifying the environmental factors that shape host–vector interactions in wading birds.

White ibis (*Eudocimus albus*) and tricolored herons (*Egretta tricolor*) are representative species with contrasting ecological and behavioral strategies. White ibis are generalist foragers that feed in groups and often exhibit nomadic movements (Heath et al., 2020), whereas tricolored herons are more specialized, forage solitarily, and display more traditional migratory patterns with less nomadism (Frederick, 2020). Despite these differences in resource use strategies, both species undertake seasonal movements that span large areas and both species are heavily reliant on wetland habitats throughout their range (Kushlan, 1981; Zhang, 2025), increasing their potential exposure to vectors and their role as hosts for vector-borne parasites (Johnson et al., 2012; Clark et al., 2016; Chahad-Ehlers et al., 2018). High prevalence of *Haemoproteus plataleae* has previously been documented in white ibis (Forrester, 1980; Coker et al., 2017; Yabsley et al., 2023), whereas tricolored herons have shown relatively low prevalence of these parasites (Telford et al., 1992; Coker et al., 2017). Studies have also confirmed the presence of infections in nestlings of tricolored herons and in both nestlings and adults of white ibis (Telford et al., 1992; Yabsley et al., 2023). In addition, infections in white ibis have been detected throughout their annual cycle (Yabsley et al., 2023). All birds in the above studies were sampled from wetland habitats in Florida, USA. Together, these data suggest that both breeding colonies and non-breeding sites may serve as locations of active transmission. However, the above studies did not investigate the effects of movement patterns or address potential differences in habitat use between the two species, which are important for understanding their exposure to vectors and the resulting patterns of haemosporidian infection. Furthermore, infections in these two species remain poorly studied outside this state, particularly in the Southeastern United States (hereafter “Southeast”), a region where both species are widely distributed and inhabit fresh- and saltwater ecosystems (Frederick, 2020; Heath et al., 2020).

To establish baseline knowledge on hemoparasites in wading birds of the Southeast and to better understand the role of bird movements in transmission, we assessed hemoparasite prevalence in wild-caught white ibis and tricolored herons from breeding colonies in coastal Alabama and used movement data to explore behavioral mechanisms of exposure. Our goals were to: (1) identify hemoparasite species in blood samples of birds and describe their relationships with closely related lineages, (2) assess differences in hemoparasite prevalence between white ibis and tricolored herons, and (3) examine prevalence differences between resident and non-resident individuals. Based on previous studies of these two wading bird species, we hypothesized that white ibis would exhibit a higher prevalence of hemoparasites than tricolored herons. In addition, we predicted that non-resident individuals would have lower hemoparasites prevalence than residents, consistent with the migratory escape hypothesis. Finally, we expected that species using freshwater habitats would show higher hemoparasites prevalence than those using saline habitats, due to the generally more favorable conditions for vector development in freshwater environments.

## 2 Materials and methods

### 2.1 Ethical approval

All capture and handling procedures followed established scientific and safety guidelines (Fair et al., 2023) and were carried out under state and federal permits, as well as University of Florida IACUC approval (Study #201910846).

### 2.2 Study area

The Gulf of America (previously known as the Gulf of Mexico; hereafter “the Gulf”) provides critical habitat for resident and migratory wading birds and serves as an important stopover along the Mississippi Flyway (Malone et al., 2021). Within this region, Mobile and Portersville Bays form broad, shallow estuarine systems along the Alabama coast. Three islands, Gaillard (30.507100, −88.035559), Marsh (30.320979, −88.222756), and Coffee (Isle aux Herbes; 30.339882, −88.255198), lie within these bays and are regularly used by white ibis and tricolored herons for foraging and nesting (May–August). The hot season of this area lasts from June to the end of September with a normal daily temperature from 18 to 32 °C. The cold season lasts from the beginning of December to the end of February with a normal daily temperature of 4–17 °C. Precipitation is frequent throughout the entire year, and the peak usually occurs in summer due to thunderstorms and hurricanes (Conner et al., 1989).

### 2.3 Capture, sampling, and tagging

We captured fledgling and adult white ibis and tricolored herons on Gaillard, Marsh, and Coffee Islands during the breeding seasons (May–August) from 2020 to 2022. To minimize disturbance, we visited each colony only once per week (Frederick and Collopy, 1989). We entered colonies after adults departed shortly after sunrise and finished capture and sampling before noon. We used a combination of dip nets, mist nets, and nest cage traps to capture birds within the colonies (Keyes and Grue, 1982; Frederick, 1986; Perkins et al., 2010). We banded all birds with a U.S. Geological Survey metal band on the left tibiotarsus and a plastic alpha-numeric band on the right tibiotarsus. We categorized captured birds as juveniles (individuals hatched during the current field season) or adults (including sub-adults and adults that were not hatched in the current season but exhibited developed morphological characteristics typical of the species; hereafter referred to as “adults”). We collected no more than 3 mL of blood from the medial metatarsal vein of each captured bird using 25- and 27-gauge needles for white ibis and tricolored herons, respectively (Owen, 2011; Coker et al., 2017). We stored blood samples in DNA/RNA Shield buffer (Zymo Research, California), kept them temporarily at −10 °C, and transferred them to a −80 °C freezer for long-term storage after the field season.

We assumed that birds would maintain the same movement patterns across years and determined their movement patterns using tracking data collected after blood sampling. We fitted birds with solar-powered Platform Transmitting Terminals (PTTs; Microwave Telemetry, Columbia, MD) to collect long-term location data for determining the migration strategy of individual birds. Depending on species and individual body size, we used transmitters of 5 g, 9.5 g, or 18 g, ensuring that each device plus harness weighed less than 3 % of the bird's body mass (Geen et al., 2019). PTTs used on tricolored herons had duty cycles of 10 h on and 48 h off, while white ibis PTTs were on for 10 h and off for 24 h. We collected location data from Service Argos (https://www.argos-system.org). We used the Douglas Filter with hybridization mode to remove locations with implausible movement rates or turning angles (Douglas et al., 2012).

During capture and processing, we monitored both ambient temperature and each bird's breathing to prevent overheating. If panting occurred, we applied 95 % ethanol to the legs and paused handling for 3 min to allow cooling (Sutherland et al., 2004). To prevent capture mortality, all birds were processed in under 30 min and released within 1.5 h of capture.

### 2.4 Hemoparasites detection and characterization

We extracted DNA from blood samples using the Quick-DNA/RNA Kit (Zymo Research, CA) and the DNeasy Blood and Tissue Kit (Qiagen, MD), following the manufacturers’ instructions. To detect haemosporidian parasites, we amplified cytochrome-b (cyt-b) sequences using a modified nested PCR protocol (Hellgren et al., 2004; Waldenström et al., 2004). The assay targets parasite DNA from *Leucocytozoon* (478 bp), *Haemoproteus* (480 bp), and *Plasmodium* (480 bp). In the first PCR step, we used primers HaemNFI and HaemNR3 to amplify sequences from all three genera. In the second step, we applied primers HaemF and HaemR2 to amplify *Plasmodium* and *Haemoproteus*, and primers HaemFL and HaemR2L to specifically amplify *Leucocytozoon* (Hellgren et al., 2004). The first PCR was performed in a 10 μl reaction containing 1 μl of genomic DNA (≥50 ng/μl), 1 μl of each primer (HaemNFI and HaemNR3; 5 μM), 5 μl of High-Fidelity PCR Master Mix with HF 2× Buffer (New England Biolabs, MA), and 2 μl of water. Cycling conditions were 98 °C for 3 min; 28 cycles of 98 °C for 30 s, 51 °C for 30 s, and 72 °C for 40 s; followed by a final extension at 72 °C for 10 min (Hellgren et al., 2004). For the second PCR, 2 μl of the first product served as template (1 μl for *Plasmodium* and *Haemoproteus* and 1 μl for *Leucocytozoon*). Reactions were prepared as above with primer pairs HaemF/HaemR2 or HaemFL/HaemR2L. Thermal cycling conditions were 98 °C for 3 min; 34 cycles of 98 °C for 30 s, 62.4 °C (*Plasmodium* and *Haemoproteus*) or 54.4 °C (*Leucocytozoon*) for 30 s, and 72 °C for 40–45 s; and a final extension at 72 °C for 10 min. PCR products from the second reaction were screened on 2 % agarose gels, where infected samples were expected to show amplicons of 478 bp (HaemF/HaemR2) or 480 bp (HaemFL/HaemR2L). Positive products were then submitted to Eurofins Genomics (Louisville, KY) for sequencing.

Only sequences exceeding 400 bp were considered as good qualitied PCR products for parasite species and lineage identification. Sequencing results were analyzed in Geneious (v2025.1, Auckland, New Zealand) and compared against GenBank (Benson et al., 2009) and the MalAvi database (Bensch et al., 2009) to assign lineages based on ≥99 % similarity. To compare the parasites detected in this study with closely related lineages, we conducted phylogenetic analyses. For phylogenetic analyses, BLAST results were used to select 30 closely related lineages from the same genus. Unique lineages were aligned using global alignment with free end gaps (65 % similarity cost matrix, 5.0/–4.0), and evolutionary relationships were inferred in Geneious with the PhyML plugin, using the Tamura–Nei substitution model and 1000 bootstrap replicates (Tamura et al., 2004). Trees were constructed with the Neighbor-Joining method and rooted with the *Plasmodium relictum* (GeneBank accession no. AY733088). Gap penalties were set to 12 for opening and 3 for extension, with sequence direction determined automatically.

### 2.5 Data analyses

Birds were classified by species and age, and movement patterns were categorized as resident or non-resident (migrants, dispersers, or nomads) based on post-breeding season movements (Gulick, 2025; Zhang, 2025). To determine whether birds were residents after they were tagged, we examined their locations after their tagging and subsequent release. We determined movement patterns only for birds that were tracked for more than three months post-release. Individuals that remained within nearby estuarine habitats of Alabama and Mississippi throughout the tracking period were classified as residents. Residency was defined as remaining within a 20-km radius of the breeding colonies; additionally, individuals that used the Mobile–Tensaw River Delta, which is located approximately 40 km north of the breeding colonies and serves as both a roosting and foraging area, were also classified as residents. All other individuals were considered non-residents (Zhang, 2025). In our analyses, we used a “non-resident” category to combine migrants, nomads, and dispersers, as white ibis in particular, show considerable plasticity in seasonal and annual movement patterns. Migrants are individuals that show predictable and seasonal departures away from a breeding area, followed by a return to the same area in a subsequent breeding season (Cottee-Jones et al., 2016). Nomads are individuals that depart the natal or breeding area and subsequently engage in extensive and unpredictable movements without a return to a previously used breeding area. Dispersers are individuals that make a directed movement away from their natal or breeding area, and subsequently establish a home range in a distinct and separate area. Thus, dispersers differ from both migrants (who return to a prior breeding area) and nomads (who do not establish a new stable home range). This classification follows a growing recognition that avian movement strategies often exist along a continuum rather than as discrete types (Cottee-Jones et al., 2016), and that accurately characterizing such variation is critical for understanding ecological processes like pathogen transmission, which depends on movement.

To assess species-level differences in freshwater and saltwater habitat use, we calculated the proportion of locations falling within each habitat type. Land cover data were obtained and reclassified using the National Wetlands Inventory (NWI) database for the United States (U.S. Fish and Wildlife Service, 2018; https://data.nal.usda.gov/dataset/national-wetlands-inventory, accessed October 15, 2025) and from the Global Lakes and Wetlands Database (GLWD) Version 2 for Central America and the Caribbean (Lehner et al., 2025). All water-associated land-cover types were reclassified as either freshwater or saltwater habitat. For each bird, we retained one location with the lowest error radius per duty cycle and calculated the proportion of locations occurring in freshwater and saltwater habitats at the species level.

We calculated parasite prevalence as the proportion of infected individuals for all tested birds, by species, by age group within a species, and by movement strategy within each species-age group. For each prevalence estimate, we obtained exact 95 % binomial confidence intervals using the Clopper–Pearson method (Clopper and Pearson, 1934). Comparisons of prevalence by age and movement pattern were evaluated using Fisher's exact tests (Upton, 1992).

## 3 Results

**Fig. 1:**
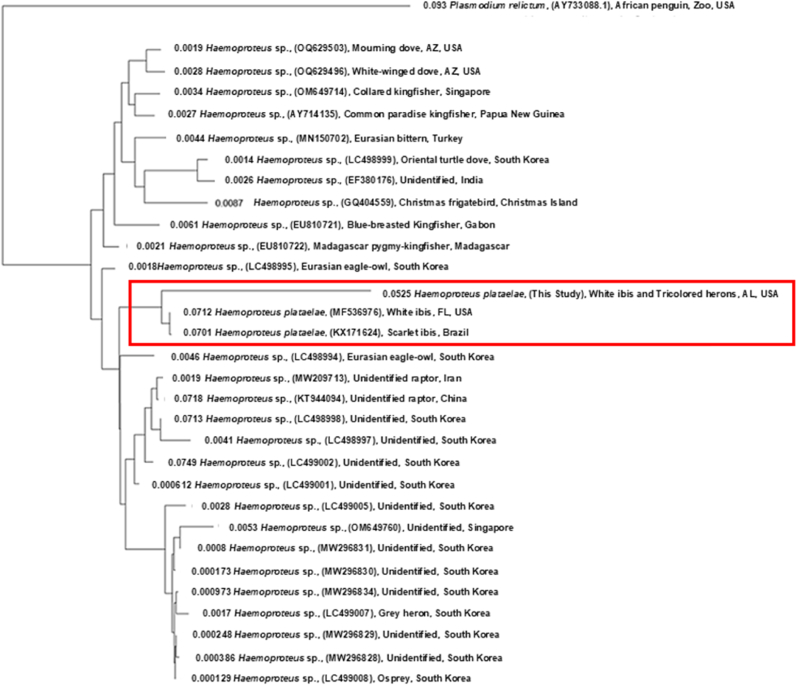
Phylogenetic relationships of *Haemoproteus plataleae* isolates from white ibis and tricolored herons sampled in breeding colonies in coastal Alabama, USA from 2020 to 2022, along with reference sequences of other *Haemoproteus* species obtained from GenBank and the MalAvi database. Record names indicate the parasite genus, GenBank locus ID, host species, and sampling location. The red rectangle highlights the sequence from this study and closely related lineages previously reported in Florida and Brazil. Numbers at nodes represent genetic distances calculated using the Tamura-Nei model, indicating evolutionary divergence between closely related lineages.

We successfully screened 164 blood samples for hemoparasites from 95 white ibis (67 juveniles and 28 adults and sub-adults) and 69 tricolored herons (45 juveniles and 24 adults). Overall, 50 birds tested positive, yielding an overall prevalence of 30.5 % (95 % CI: 23.5–38.1 %) across species. Overall, haemoparasite prevalence was 42.1 % (40 of 95) in white ibis and 14.5 % (10 of 69) in tricolored herons (Table 1). All infections were identified as *Haemoproteus plataleae*. Of the 50 samples sequenced from test-positive birds, 26 produced high-quality sequences longer than 400 bp. BLAST searches in GenBank showed that these sequences matched a single lineage (GeneBank accession no. h**EUDRUB01**), previously documented in a captive scarlet ibis (*Eudocimus ruber*) in Brazil (Chagas et al., 2017) and in free-ranging white ibis and green herons (*Butorides virescens*) in South Florida (Fig. 1, Yabsley et al., 2023).

**Table 1:** Prevalence of haemosporidian parasites in white ibis and tricolored herons sampled in breeding colonies in Coastal Alabama, USA from 2020 to 2022.

Species	Age	No. Positive/No. Sampled (%)	95 % Confidence Interval (%)
White ibis	Adult	19/28 (67.9 %)	47.6–84.1
	Juvenile	21/67 (31.3 %)	20.6–43.8
Tricolored heron	Adult	2/24 (8.3 %)	1.0–27.0
	Juvenile	8/45 (17.8 %)	8.0–32.1
Total		50/164 (30.5 %)	23.5–38.1

White ibis had a significantly higher prevalence of hemoparasites compared to tricolored herons (Fisher's exact test, p = 0.00014, odds ratio = 4.25, 95 % CI: 1.87–10.49; Table 1). Age-related comparisons showed that adult ibis were more likely to be infected than juveniles (Fisher's exact test, p = 0.0014, odds ratio = 0.22, 95 % CI: 0.07–0.61; Table 1), whereas tricolored herons exhibited no difference between age classes (Fisher's exact test, p = 0.475, odds ratio = 2.35, 95 % CI: 0.42–24.70; Table 1).

Among the birds tracked with satellite transmitters (n = 38 for white ibis; n = 24 for tricolored herons), 35 white ibis (14 juveniles and 21 adults) were classified as non-residents (migrants, dispersers, or nomads), while three adults were identified as residents. In contrast, 18 tricolored herons (7 juveniles and 11 adults) were classified as non-residents, and six (1 juvenile and 5 adults) remained as residents (Table 2). For juvenile white ibis, no residents were sampled, precluding any comparison between movement strategies between age groups. For juvenile tricolored herons, the single resident tested negative, whereas 57.1 % (4 of 7) of non-residents were infected. Because only one resident juvenile was sampled, statistical comparison between movement strategies was uninformative (Table 2). Among adult white ibis, 100 % (3/3) of resident individuals and 67 % (14/21) of non-resident individuals tested positive, however, this difference was not significant (Fisher's exact test, p = 0.53). Due to all resident adults testing positive, the odds ratio was infinite, thereby confidence intervals were inestimable (Table 2). Among adult tricolored herons, none of the five residents tested positive, whereas 9.1 % (1 of 11) of non-residents were infected; this difference was not significant (Fisher's exact test, p = 1.00; Table 2).

**Table 2:** Prevalence of haemosporidian parasites in tracked white ibis and tricolored herons sampled in breeding colonies in Coastal Alabama, USA from 2020 to 2022.

Species	Age	Movement Strategy	No. PCR Positive/No. Tested (%)	95 % Confidence Interval (%)
White ibis	Juvenile	Resident	N.A	N.A
		Non-Resident	6/14 (42.9 %)	17.7–71.1
	Adult	Resident	3/3 (100.0 %)	29.2–100.0
		Non-Resident	14/21 (66.7 %)	43.0–85.4
Tricolored heron	Juvenile	Resident	0/1 (0.0 %)	0.0–97.5
		Non-Resident	4/7 (57.1 %)	18.4–90.1
	Adult	Resident	0/5 (0.0 %)	0.0–52.2
		Non-Resident	1/11 (9.1 %)	0.2–41.3
Total			28/62 (45.2 %)	32.5–58.3

**Fig. 2:**
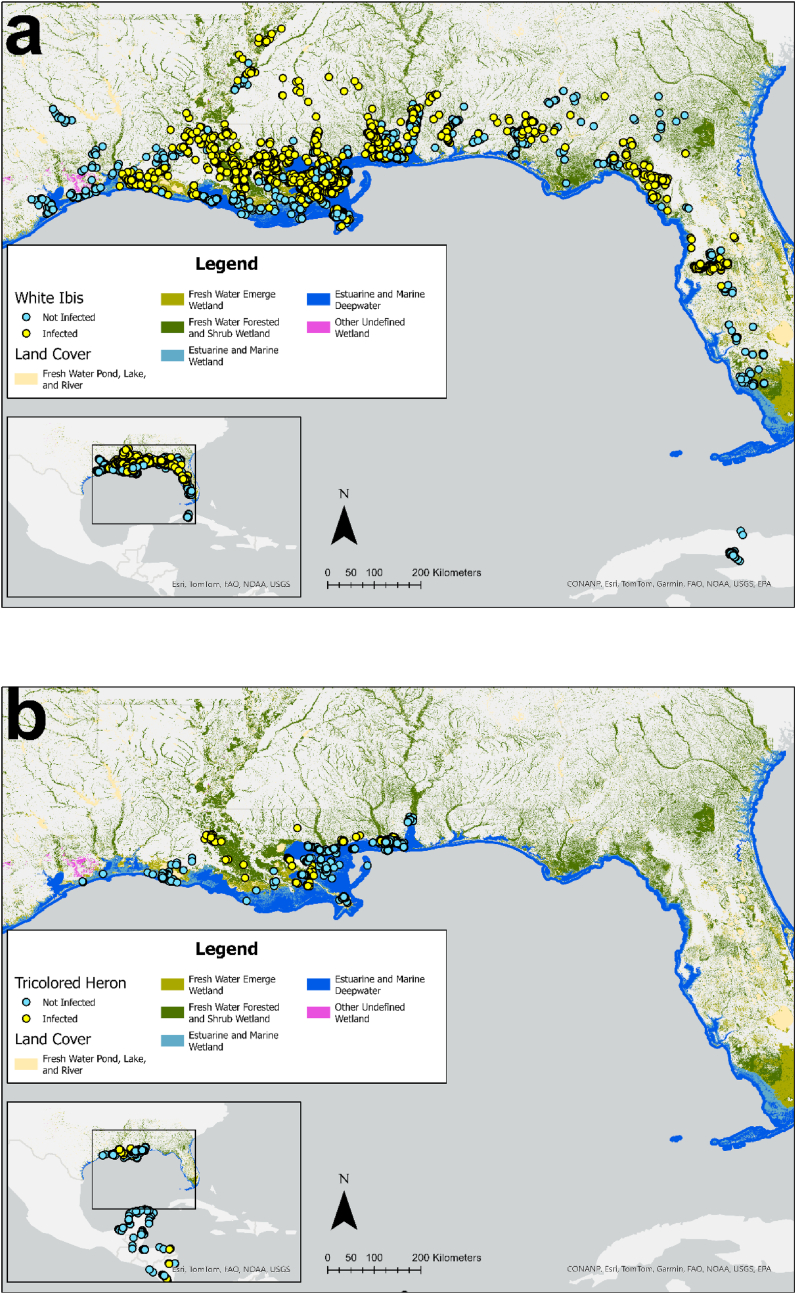
Tracked locations of white ibis (a) and tricolored herons (b) sampled from breeding colonies in coastal Alabama, USA during 2020–2022. One location point per bird per duty cycle was randomly selected from non-breeding season data for display on the map. Points are colored by infection status (hemoparasite infected vs. not infected). Land cover data are shown for the southeastern United States.

**Fig. 3:**
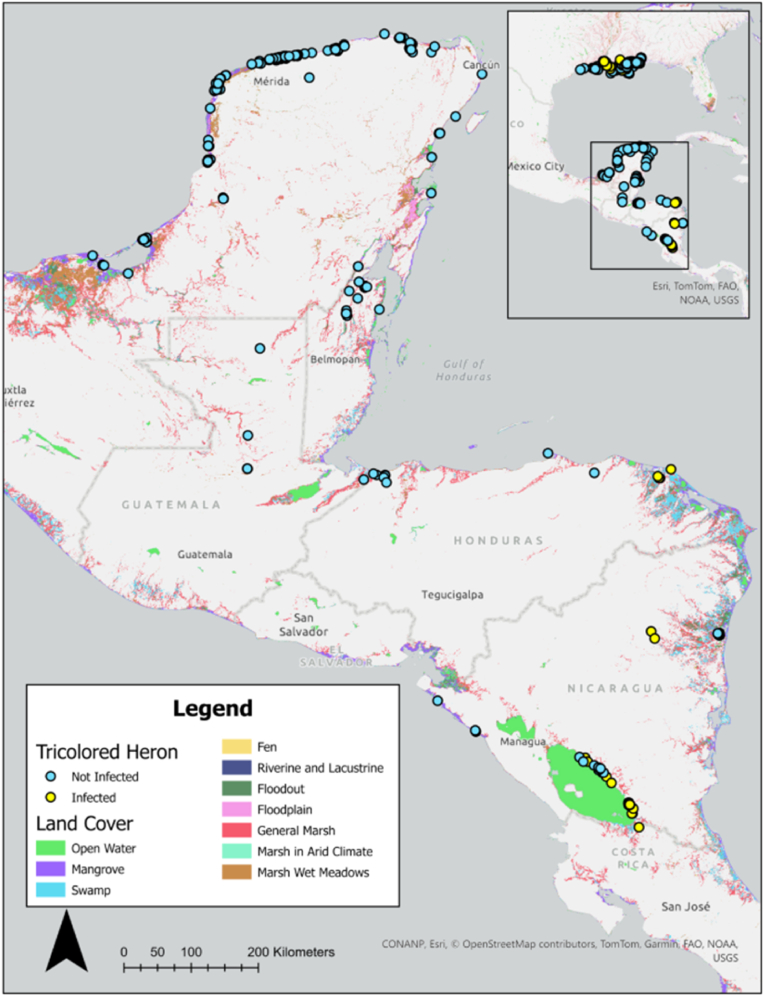
Tracked locations of tricolored herons sampled from breeding colonies in coastal Alabama, USA during 2020–2022. One location point per bird per duty cycle was randomly selected from non-breeding season data for display on the map. Points are colored by infection status (hemoparasite infected vs. not infected). Land cover data are shown for Central America and the Caribbean region.

After departing their breeding colonies, white ibis wintered throughout the southeastern United States, using areas in Florida, Alabama, Mississippi, Louisiana, and Texas; one white ibis wintered in Cuba. After departing the breeding colonies, white ibis exhibited varying degrees of nomadic behavior, moving extensively across large areas during the non-breeding season. Individuals used a combination of inland freshwater, coastal saltwater, as well as other habitats (Fig. 2a; Zhang, 2025). 60.3 % of white ibis non-breeding locations were in freshwater habitats, and 39.7 % were in saltwater habitats (including the single white ibis that wintered in Cuba). Infected white ibis wintered in Florida, Alabama, Mississippi, Louisiana, and Texas (Fig. 2a). In contrast, tricolored herons primarily used coastal saltmarsh habitats after departing from their breeding colonies, including estuaries in coastal Alabama, Mississippi, Louisiana, and Texas (Fig. 2b). Some individuals exhibited trans-Gulf migratory behavior, wintering in several Central American countries. Once they reached their wintering sites, they generally exhibited relatively small home ranges and continued to use coastal saltmarsh habitats (Fig. 3; Zhang, 2025). Among the infected tricolored herons, one adult wintered in Lake Nicaragua (Lago Cocibolca), one stopped transmitting while transiting the Gulf, and the remaining individuals were last tracked in either Mississippi or Louisiana. Before trans-Gulf migration, 14.5 % of tricolored heron non-breeding locations were in freshwater habitats, and 85.5 % were in saltwater habitats. After migration, 34.6 % of locations occurred in freshwater habitats and 65.4 % in saltwater habitats.

## 4 Discussion

This study is the first to compare haemosporidian infections between two ecologically distinct wading bird species, the white ibis and the tricolored heron, and incorporating movement patterns and wintering location data from the sampled birds. We also provide the first evidence of hemoparasite infections in white ibis and tricolored herons from coastal breeding colonies in the southeastern United States outside of Florida. For both wading bird species, we detected only one hemoparasite species, *Haemoproteus plataleae*, which has previously been reported in ibis and spoonbill species as a wading bird specialist (de Mello, 1935; Forrester, 1980). Genetic analysis confirms that all samples matched the previously reported lineage (GeneBank accession no. **hEUDRUB01**) from Florida (Yabsley et al., 2023). The similarity to a lineage previously reported in Florida suggests that this parasite species may exhibit low population genetic structure throughout the Southeast. This pattern may result from frequent parasite exchange among host populations facilitated by bird movements (Gil-Vargas and Sedano-Cruz, 2019; Humphries et al., 2019). For example, although no individuals tracked in our study were documented visiting the sites in south Florida described by Yabsley et al. (2023), they may share undetected stopover sites and wintering areas, and other wading bird species could also contribute to parasite transmission among shared sites within and across regions.

In our study, prevalence differed markedly between species: white ibis showed substantially higher infection rates than tricolored herons. In the Alabama breeding colonies, the overall prevalence in white ibis was comparable to earlier reports from Florida (Yabsley et al., 2023), whereas tricolored herons showed relatively low infection levels, also consistent with previous findings from Florida (Telford et al., 1992). These interspecific differences may be explained by the morphological characteristics and behaviors of the hosts (Valkiunas, 2004; Fecchio et al., 2020). For example, ibis species possess larger patches of bare skin on the head around the eyes compared to the more feather-covered skin in the same area in herons, potentially providing easier access for biting vectors (Darbro and Harrington, 2007). In addition, differences in the intensity of social behavior between the two species may be a contributing factor. White ibis exhibit large-flock foraging and communal roosting behaviors (Eiserer, 1984; Petit and Bildstein, 1987), which could further enhance transmission by attracting vectors to groups of hosts (Laughlin et al., 2019). By contrast, tricolored herons are generally considered less social (Frederick, 2020). Although they are sometimes observed in flock foraging or roosting (Caldwell, 1981), their flock sizes are much smaller than those of white ibis. Finally, vector specialization may influence infection prevalence (Doussang et al., 2021), though identification of the main wading bird vectors and whether they exhibit host-specific preferences requires further study. Despite the factors mentioned above, the most important determinant of the observed differences in infection prevalence may be bird movements, as it determines where individuals go during their annual cycle and which habitats they use, thereby influencing their exposure to vectors.

Infections were detected in both resident and non-resident white ibis, including adults and juveniles. All 14 juvenile white ibis exhibited non-resident behavior, whereas some adults (3 of 24) exhibited resident behavior; this pattern is likely because juveniles are undergoing natal dispersal (Heath et al., 2020). Although not all non-resident white ibis showed classic migratory behavior, they did undertake directed, long-distance movements away from the natal or breeding colonies. These movements are ecologically comparable to tricolored heron migration in their potential to enable individuals to move away from areas of high vector abundance. These findings, together with evidence of infections across both younger and older age groups in the full sample set of both species, suggest that transmission can occur at both breeding colonies and non-breeding sites. Small and dissimilar sample sizes in this study limited comparisons of prevalence and constrained our ability to test the hypotheses that birds who leave their breeding sites either escape or acquire parasites at non-breeding sites (Altizer et al., 2011). Meanwhile, several white ibis that survived for multiple years eventually shifted their movement strategies between dispersal and migratory behavior, making it more difficult to directly test these hypotheses. Finally, some individuals classified as residents were tracked for only a limited period, and it is possible that these birds died or their transmitters failed prior to classification of their movement behavior. Nonetheless, the high prevalence observed in non-resident white ibis (66.7 %), together with the finding that all infected tricolored herons were migrants, suggests the potential these birds fit the migratory exposure hypothesis, which proposes that large-scale movement increases opportunities for encountering parasites (Altizer et al., 2011).

Specifically, we found that movements of white ibis from coastal breeding colonies in Alabama were largely confined to the southeastern United States rather than extending into Central America, despite the fact that some migrated directly to defined wintering sites, while others showed nomadic behaviors, frequently shifting locations and occupying relatively large areas throughout the non-breeding season. During the annual cycle, white ibis used a combination of freshwater and saltwater habitats with many individuals dispersing farther inland to utilize rivers, ponds, lakes, and created wetlands (including commercial aquaculture sites) embedded primarily in agriculture habitats (Gulick, 2025; Zhang, 2025). In general, freshwater habitats may provide more favorable conditions for vector development (Turnipseed, 2017), offering greater vegetation cover and less salt stress on larval stages (Singh et al., 2022), thereby enhancing survival and growth of vectors of haemosporidians. In addition, white ibis may experience higher exposure to vectors in aquaculture habitats such as crawfish farms, where red swamp crayfish (*Procambarus clarkii*) prey on dragonfly nymphs (*Aeshna* spp.), thereby reducing predation pressure on mosquito larvae and potentially increasing their abundance (Bucciarelli et al., 2019). Meanwhile, some infected individuals also used large estuarine areas in Louisiana, which appears to contradict the explanation that freshwater habitats increase vector exposure. However, saline estuaries have unique landscape characteristics that can support high vector densities (Weller and Bossart, 2017; Aker et al., 2023), possibly because they contain tall, dense vegetative structure (e.g., *Juncus* sp., *Phragmites* sp., *Spartina* sp., *Typha* sp.; Visser et al., 2017) that may allow salt-tolerant vector species to persist in these habitats (Ramasamy and Surendran, 2012). Finally, not all Diptera species function as competent vectors (Courtney et al., 2017). Therefore, the general concept that freshwater habitats are more favorable for vectors reflects only that these habitats have a greater probability of supporting competent vector species; it does not necessarily guarantee that competent vectors were more abundant in freshwater habitats in our study. Information on vector competence and distribution would allow direct testing of this hypothesis, but such data were beyond the scope of our study. In addition, resident white ibis that remain in the colony areas may also play a role in parasite transmission. According to classical transmission theory, seasonal pulses of vector abundance and host aggregation can drive rapid increases in prevalence (Anderson and May 1978), and resident ibis that remain near colonies throughout the year may sustain local transmission cycles between breeding seasons (Lachish et al., 2011). These residents could serve as reservoirs that maintain parasites through periods of low host density, allowing for re-seeding of infections when colony numbers rise in the next breeding season.

Infections were detected in non-resident tricolored herons, including juveniles and adults. Seven of eight juveniles and 11 of 16 adults exhibited non-resident behavior, and as in white ibis, the higher proportion of non-resident juveniles among tricolored herons is likely attributable to natal dispersal (Frederick, 2020). We found that tricolored herons exhibited movements not only to estuaries along the coast of the Southeast but also across the northern Gulf, with some individuals reaching Central America and the Caribbean, where they spent most of their annual cycle in saltmarsh habitats (Zhang, 2025); which could explain the low prevalence of hemoparasites in this species (Adler and Courtney, 2019). In addition, their long-distance movements into the tropics raises the possibility that herons may act as occasional vectors for introducing novel parasite lineages into the southeastern United States or vice versa, even if the overall contribution to prevalence is modest (Santiago-Alarcon et al., 2012). The lineage detected in this study has also been reported, in addition to Florida, from a scarlet ibis kept in a zoo in Brazil (Chagas et al., 2017). Although the transmission route between the Southeast and Brazil remains unknown, it is possible that long-distance migratory movements facilitate parasite exchange, either by introducing parasites into breeding colonies in the Southeast or by spreading them southward into Central America. However, testing this hypothesis is beyond the scope of this study.

In conclusion, there seems to be low population genetic structure for *Haemoproteus plataleae* on wading bird species in the Southeast. White ibis showed a higher prevalence of haemosporidian infection than tricolored herons, consistent with our prediction and results from previous studies (Yabsley et al., 2023; Telford et al., 1992). We could not directly test the migratory escape hypothesis because of imbalanced sample sizes across age classes and between resident and non-resident groups. Instead, we suggest a possible transmission role in which white ibis function primarily as local amplifiers of parasites, maintaining transmission along the northern Gulf region, whereas tricolored herons may serve as occasional long-distance dispersers facilitating parasite exchange at lower frequency. The greater use of freshwater habitats by white ibises, together with their higher prevalence of infection, compared with the greater use of estuarine habitats and lower prevalence of infection in tricolored herons, is consistent with the hypothesis that species using freshwater habitats exhibit higher haemosporidian prevalence than those using saline habitats. These results highlight the complex interactions among movement strategies, species-specific traits, and host-parasite dynamics in shaping infection prevalence. They also emphasize the need to account for ecological factors such as habitat use and vector exposure when studying avian infectious disease systems. Future research that addresses identifying competent vectors and modeling vector and host distributions at finer spatial scales, fine-scale habitat use analyses within known high use sites across seasons could improve understanding of the transmission processes.

## CRediT authorship contribution statement

**Ke Zhang:** Writing – original draft, Methodology, Investigation, Data curation, Conceptualization. **Samantha M. Wisely:** Writing – review & editing, Supervision, Resources, Methodology, Conceptualization. **Chris K. Gulick:** Writing – review & editing, Investigation. **Abby N. Powell:** Writing – review & editing, Supervision, Project administration, Methodology, Investigation, Funding acquisition, Data curation, Conceptualization.

## Conflict of interest

The authors declare no conflict of interest.
